# Agreement and Discrepancy on Emotional and Behavioral Problems Between Caregivers and HIV-Infected Children and Adolescents From Uganda

**DOI:** 10.3389/fpsyt.2019.00460

**Published:** 2019-07-11

**Authors:** Leigh L. van den Heuvel, Jonathan Levin, Richard S. Mpango, Kenneth D. Gadow, Vikram Patel, Jean B. Nachega, Soraya Seedat, Eugene Kinyanda

**Affiliations:** ^1^Department of Psychiatry, Faculty of Medicine and Health Sciences, University of Stellenbosch, Cape Town, South Africa; ^2^School of Public Health, Faculty of Health Sciences, University of the Witwatersrand, Johannesburg, South Africa; ^3^Mental Health Project, MRC/UVRI Uganda Research Unit on AIDS/Senior Wellcome Trust Fellowship, Entebbe, Uganda; ^4^Department of Psychiatry, Stony Brook University, New York, NY, United States; ^5^Department of Global Health and Social Medicine, Harvard Medical School, Massachusetts, MA, United States; ^6^Departments of Epidemiology, Infectious Diseases and Microbiology, University of Pittsburgh Graduate School of Public Health, Pittsburgh, PA, United States; ^7^Departments of Epidemiology and International Health, Johns Hopkins Bloomberg School of Public Health, Baltimore, MD, United States; ^8^Department of Medicine and Centre for Infectious Diseases, Faculty of Medicine and Health Sciences, Stellenbosch University, Cape Town, South Africa; ^9^Department of Psychiatry, Makerere College of Health Sciences, Kampala, Uganda; ^10^Department of Population Health, London School of Hygiene and Tropical Medicine, London, United Kingdom

**Keywords:** children, adolescents, HIV, emotional problems, behavioral problems, caregiver report, self-report, discrepancy

## Abstract

**Background:** HIV-infected children and adolescents (CA-HIV) face significant mental health challenges related to a broad range of biological and psychosocial factors. Data are scarce on the agreement and discrepancy between caregivers and CA-HIV regarding emotional and behavioral problems (EBPs) in CA-HIV.

**Objectives:** We determined agreement between self- versus caregiver- reported EBPs and describe factors associated with informant discrepancy among caregiver–youth dyads who participated in the “Mental health among HIV-infected CHildren and Adolescents in KAmpala and Masaka, Uganda” (CHAKA) study.

**Methods:** In a cross-sectional sample, caregiver-reported EBPs were assessed with the Child and Adolescent Symptom Inventory-5 (CASI-5), and self-reported problems were evaluated with the Youth Inventory-4 (YI-4) in 469 adolescents aged 12–17 years and the Child Inventory-4 (CI-4) in 493 children aged 8–11 years. Adolescents were questioned about experiences of HIV stigma. Caregiver psychological distress was assessed with the Self-Reporting Questionnaire (SRQ-20). Linear regression models were applied to identify variables associated with discrepancy scores.

**Results:** Self-reported emotional problems (EPs) were present in 28.8% of adolescents and 36.9% of children, and 14.5% of adolescents self-reported behavioral problems (BPs). There was only a modest correlation (*r* ≤ 0.29) between caregiver- and CA-HIV-reported EBPs, with caregivers reporting more EPs whereas adolescents reported more BPs. Informant discrepancy between adolescents and caregivers for BPs was associated with adolescent age and caregiver’s employment and HIV status. Among adolescents, EP discrepancy scores were associated with adolescent’s WHO HIV clinical stage, caregiver level of education, and caregivers caring for other children. Among children, EP discrepancy scores were associated with child and caregiver age, caregiver level of education, and caregiver self-rated health status. HIV stigma and caregiver psychological distress were also associated with discrepancy, such that adolescents who experienced HIV stigma rated their EPs as more severe than their caregivers did and caregivers with increased psychological distress rated EBPs as more severe than CA-HIV self-rated.

**Conclusions:** EBPs are frequently endorsed by CA-HIV, and agreement between informants is modest. Informant discrepancy is related to unique psychosocial and HIV-related factors. Multi-informant reports enhance the evaluation of CA-HIV and informant discrepancies can provide additional insights into the mental health of CA-HIV.

## Introduction

Of the 36.7 million people living with HIV in 2016, 52.9% (19.4 million) were residing in Eastern and Southern Africa (1). In Uganda in 2016, there were 1.4 million people living with HIV, of which 130,000 were under the age of 15 years (2). Despite the high health care burden of HIV in Sub-Saharan Africa, the majority of studies evaluating the mental health of HIV-infected children and adolescents (CA-HIV) have been conducted in developed regions (3). Generally, studies show that CA-HIV have high rates of medical and psychiatric morbidity (3–7). An earlier review of psychiatric disorders in CA-HIV based on the *Diagnostic and Statistical Manual of Mental Disorders* (*DSM*) nosology reported an average prevalence across studies of 28.6% for attention deficit hyperactivity disorder (ADHD), 24.3% for anxiety disorders, and 25.0% for depression (8). When rates of mental health problems are compared to other high-risk groups, such as HIV-exposed but uninfected youth or youth from HIV-affected households (e.g., AIDS orphans or HIV-infected caregivers), results tend to be more mixed (3, 6, 7, 9–12). Studies from African countries with a high prevalence of HIV have also found clinically significant rates of mental health problems. For example, in a Kenyan study, 48.8% of CA-HIV received a *DSM-IV*-based diagnosis (13), and an earlier cross-sectional Ugandan study in antiretroviral (ARV) naive adolescents found that over half (51.2%) self-reported significant psychological distress, and anxiety (45.6%) and depression (40.8%) were the most common International Classification of Diseases (ICD) psychiatric disorders (14).

CA-HIV from both high-income and resource-limited settings face increased mental health challenges that are related to a broad range of biopsychosocial factors (3, 15), such as overall health status, cognitive functioning, caregiver general health and mental health status, stressful life events, neighborhood stressors, and a lack of social support (3). HIV clinical disease factors, such as CD4 cell count and viral load, have not consistently been linked to poorer mental health outcomes; some studies have reported relations between indicators of HIV progression and mental health problems (16–19), whereas others have found no clear links (5, 10, 20, 21). Further, the presence of psychiatric disorders in CA-HIV has been associated with increased risk behaviors, including substance abuse, treatment non-adherence, and early-onset sexual intercourse (22–25). Stigma, related to being HIV positive, is another factor impacting the well-being of CA-HIV. Meta-analyses have demonstrated that HIV-related stigma is associated with various negative outcomes, including adverse mental health outcomes, such as increased depression, anxiety, and psychological distress (26). Similarly, studies in Africa have demonstrated an association between HIV stigma and increased mental health problems in both adult and adolescent samples (23, 27–29).

There is general consensus that a thorough assessment of child and adolescent mental health problems requires integrating information from various sources, including caregivers, educators, health care providers, and youth self-report (30, 31). Children and adolescents can provide information that may not be known to caregivers such as internal experiences (e.g., thought content, affect) or symptoms and behaviors that occur in contexts where caregivers are not present, such as school or peer interactions (32, 33). Certain problems may be underreported by caregivers, such as abuse and neglect by caregivers, and youth may keep some behaviors hidden from caregivers such as substance use or antisocial behaviors (33). Furthermore, youth self-report can assist in improving accuracy of certain diagnoses, in treatment planning, and in determining the reliability of caregiver information (33). Youth self-report can also provide unique insights into CA-HIV. For instance, in one study, CA-HIV reported elevated depression scores compared to HIV-negative youth, whereas there were no differences by youth HIV status for internalizing or externalizing problems according to caregiver reports (34). Furthermore, a multisite study in CA-HIV and HIV-affected youth found that having received prior mental health interventions was associated with caregiver-reported emotional and behavioral problems (EBPs), but not with youth self-reported EBPs, suggesting that youth self-report may be overlooked in routine practice (35).

Overall, agreement between caregiver-reported and youth self-reported problems is modest at best (32, 36, 37). Studies in CA-HIV have similarly reported low agreement between caregiver and youth self-report (5, 7). The agreement between reports from different informants, while providing information regarding different perspectives and contexts, does not reflect inter-rater reliability (30, 31, 36). For example, research has shown that the correlation between informants does not change much over time, whereas the correlation within informants does change substantially over time (36).

Research has consistently demonstrated discrepancies between different informants about child EBPs (38). As each informant provides a unique contribution to the assessment of EBPs, disagreements that may arise from informant reports can provide more information than when informants agree (36). Discrepancies between caregiver and youth report may provide additional information above each report alone, such as contextual variations in symptoms and treatment response, the individual characteristics of the informants, and features of the caregiver–child relationship (39). For instance, discrepancies in parent- and child self-reported social functioning among youth with autism spectrum disorder provided additional information about parental self-efficacy, youth psychopathology, and treatment response and predicted outcomes better than parent or youth self-report alone (39). Furthermore, another study demonstrated that teacher–adolescent pretreatment discrepancy about prosocial behaviors predicted post-treatment caregiver-rated improvements, whereas the actual ratings of the teachers and adolescents individually did not. Adolescents showed greater improvements according to caregiver report if teachers rated their prosocial behaviors as better than adolescents did themselves, again demonstrating the value of multi-informant report (40).

Discrepancies between caregiver and youth self-reports have been associated with the development of child psychopathology, caregiver stress, and problems in the caregiver–child relationship, although there is no clear patterning between informant characteristics and discrepancies (38). Caregiver psychopathology, and in particular depression, has been one of the most consistent factors associated with informant discrepancy of EBPs (38). Caregivers who are depressed or anxious rate their children’s EBPs as more severe than other informants, such as teachers and youth themselves (38). Furthermore, a meta-analysis revealed that the association between maternal depression and child EBPs was significantly greater if maternal report was used as compared to child self-report, a combination of mother and child self-report, or reports from teachers or others (41). Inconsistencies in the association between caregiver psychological distress and informant discrepancy pertain mainly to specifics, such as whether anxiety or depression is the primary factor contributing to discrepancy or how the child’s age and gender influence the outcomes (38).

EBPs are associated with adverse outcomes among CA-HIV, yet the source of information used to establish their presence is often not taken into consideration. Discrepancy between informants can provide more information than informant reports alone (36, 39). Moreover, informant discrepancies have also been associated with poorer treatment outcomes (42–44). Yet, informant discrepancy of EBPs have not been evaluated among CA-HIV, a group facing additional challenges, such as parental illness, orphanhood, HIV stigma, and HIV disease and treatment-related factors (3, 15, 26). Caregivers of HIV-infected children also face increased challenges, such as financial strain, food insecurity, parenting stress, anxiety, depression, and difficulties pertaining to accessing health care services and treatment adherence (45–49). Studies have also demonstrated discrepancies between caregiver and youth-reported barriers to ART adherence, thus further demonstrating the value of assessing informant discrepancy among CA-HIV (50). Evaluating informant discrepancy of EBPs in CA-HIV can provide further insights into the factors affecting mental health outcomes in CA-HIV and thus help inform treatment strategies.

The primary objective of this study was to better understand the clinical correlates of informant discrepancy between caregiver-reported and CA-HIV self-reported *DSM-5*-referenced EBPs. To the best of our knowledge, this is the first study to examine the clinical implications of informant discrepancy among CA-HIV. The study sample comprised youth who were participating in the *Mental health among HIV-infected CHildren and Adolescents in KAmpala and Masaka, Uganda* (CHAKA) study. Specifically, we describe relations between informant discrepancy and a range of sociodemographic and HIV-related factors for the symptoms of a number of common child and adolescent EBPs. Additionally, we investigate the association of HIV stigma and caregiver psychological distress with informant discrepancy. We also report on the prevalence of self-rated EBPs as compared to caregiver-rated EBPs and assessed the level of agreement. Based on existing research (36, 51), we hypothesized that CA-HIV would self-rate a greater number and severity of EBPs than caregivers.

## Materials and Methods

### Study Design

The CHAKA study assessed the prevalence of, and factors associated with, psychiatric disorders among CA-HIV. Participants were recruited between January 2014 and June 2015. Published manuscripts addressing other research questions emanating from this study can be reviewed for further details (52–55). The study was conducted in accordance with the Declaration of Helsinki and ethical approval was obtained from the Uganda Virus Research Institute’s Research and Ethics Committee, the Ethics Committee of the London School of Hygiene and Tropical Medicine, and the Uganda National Council of Science and Technology.

### Setting

A sample of 1,339 child/adolescent–caregiver dyads was recruited from five HIV clinics in central and southwestern Uganda, three in the rural Masaka district (the AIDS Support Organisation clinic, Kitovu Mobile AIDS organisation, and the Uganda Cares clinic) and two in the urban Kampala City Council (Joint Clinical Research Centre and Nsambya Homecare Department). Eligible participants were recruited from each study site consecutively until the required sample size was attained. An equal number of 268 dyads was planned for recruitment at each site. Interviews were conducted in partitioned tents that were erected at each of the study sites to ensure privacy and limit distraction.

### Participants

CA-HIV between 5 and 17 years of age with caregivers older than 17 years of age were included. Additionally, participants were included if both caregivers and CA-HIV could speak English or Luganda (the local language spoken in the study areas), and they remained in the study’s geographical area for the subsequent 12 months. Participants were excluded if they were concurrently enrolled in another study, if they were unwell and in need of immediate medical attention, and if they did not understand the study instruments for any reason. Furthermore, to be able to address the objectives of this study, we only included CA-HIV who had completed the self-report measures for EBPs. Eligible study participants provided written informed consent (caregiver) and assent (CA-HIV) after explanation of the study objectives and procedures. No CA-HIV were enrolled without their assent, and all participants were informed that they could withdraw without prejudice at any time. In the majority of cases, the parents provided informed consent for participation of the CA-HIV, but in cases where the primary caregivers were not parents, the guardians of the CA-HIV provided the informed consent. Approximately 2% of participants assessed for eligibility were not included due to factors such as caregiver refusal, CA-HIV refusal, inability to contact the caregiver to obtain consent, and ongoing participation in another study.

### Procedure

The assessment battery comprised structured, standardized, and locally translated instruments. Measures not previously used in Uganda were forward and back translated and locally adapted and piloted before use (52, 55). Assessments were administered by trained psychiatric nurses and psychiatric clinical officers and supervised by a psychiatrist and a clinical psychologist. All measures used were read to participants to accommodate for variation in reading level. Participants diagnosed with putative psychiatric disorders were provided with psychoeducation and referred to local mental health care services. A demographic questionnaire was designed to obtain sociodemographic information of caregivers and CA-HIV [e.g., age, gender, employment status, highest level of education (HLOE), caregiver relationship to child] and a medical questionnaire to obtain data regarding caregiver and child health status (e.g., nadir CD4, current CD4, ART status, caregiver HIV status).

#### Measures

##### Emotional and Behavioral Problems

Caregivers completed the parent version of the *Child & Adolescent Symptom Inventory-5* (*CASI-5*) (56), which includes the symptoms of *DSM-5* psychiatric disorders among youth aged between 5 and 18 years old. Symptoms are rated on a four-point Likert scale (0—*never*, 1—*sometimes*, 2—*often*, 3—*very often*) with an impairment rating (rated on the same Likert scale as symptoms) for each disorder. The CASI-5 can be utilized to obtain a *symptom cutoff score* (number of symptoms required for a *DSM-5* diagnosis rated “2” or higher), an *impairment cutoff score* (impairment rated “2” or higher, regardless of number of symptoms), a *clinical cutoff score* (has to fulfill both symptoms cutoff and impairment cutoff scores), and a *symptom severity score* (dimensional model). The symptom severity scores are calculated by adding the individual ratings of each of the symptoms for each disorder. We evaluated for the presence of EBPs utilizing the symptom cutoff score (see **Table 1**) and used the severity scores to calculate discrepancy on EBPs. The CASI has been used in hundreds of studies (57), including in HIV-positive youth (7), and has demonstrated satisfactory psychometric properties, including internal consistency (Cronbach’s α between 0.45 and 0.92), test–retest reliability (*r* > 0.65), and convergent, divergent, and discriminant validity in various settings (58–61). The CASI-5 was adapted for use in the local Ugandan setting (52) and internal consistency was satisfactory (Cronbach’s α between 0.70 and 0.85) (55).

**Table 1:** Emotional and behavioral problems as assessed with rating scales.

Problems assessed	CASI-5^a^	YI-4^b^	CI-4^c^
*Behavioral problems*			
Attention deficit hyperactivity disorder (ADHD)	AS	AS	−
Oppositional defiant disorder (ODD)	AS	AS	−
Conduct disorder (CD)	AS	AS	−
Substance use disorder (SUD)	SQ(s)	SQ(s)	−
*Emotional problems*			
Anxiety disorders			
Generalized anxiety disorder (GAD)	AS	AS	AS
Specific phobia	SQ(s)	SQ(s)	SQ(s)
Panic disorder	SQ(s)	SQ(s)	−
Social anxiety disorder (SAD; social phobia)	AS	SQ(s)	SQ(s)
Separation anxiety disorder	AS	SQ(s)	AS
Mood disorders			
Major depressive episode (MDE)	AS	AS	AS
Persistent depressive disorder (dysthymia)	AS	AS	AS
Related disorders			
Posttraumatic stress disorder (PTSD)	SQ(s)	SQ(s)	SQ(s)
Somatic symptom disorder	SQ(s)	SQ(s)	SQ(s)

a Administered to caregivers.

b Administered to adolescents (ages 12–17 years).

c Administered to children (ages 8–11 years).

AS; all symptoms; CASI-5, Child & Adolescent Symptom Inventory-5; CI-4, Child Inventory-4; SQs, screening question(s); YI-4, Youth’s Inventory-4.

Adolescents between 12 and 18 years old completed the *Youth’s Inventory-4* (*YI-4*) (62), which is a self-report measure of *DSM*-referenced symptoms. The YI-4 comprises 120 items that correspond to items in the CASI-5 and is rated and scored in a similar way to the CASI-5. The YI-4 has demonstrated satisfactory internal consistency (Cronbach’s α between 0.66 and 0.87) and test–retest reliability (*r* between 0.54 and 0.92) and aligns well with other self-report measures and clinical diagnoses (33). Internal consistency in this study was also fair (Cronbach’s α between 0.49 and 0.88) (55).

*The Child Self-Report Inventory-4* (*CI-4*) (63) is a parallel self-report measure for use with children aged between 8 and 11 years and includes 34 items that are phrased and rated similarly to the YI-4 and CASI-5, but does not include an impairment rating. The CI-4 rates only for EPs (**Table 1**), and thus, for the child sample, results are limited to EPs. Formal validation studies have not yet been published regarding the CI-4, although it has been used in other studies of HIV-positive children (61, 64). In this sample, the internal consistency of disorders assessed (Cronbach’s α between 0.62 and 0.79) as well as the full scale (Cronbach’s α = 0.89) was satisfactory.

##### Caregiver Psychological Distress

*The Self-Reporting Questionnaire* (*SRQ-20*) (65) is a brief measure developed by the WHO to screen for common mental problems, such as depression and anxiety, in developing countries. Respondents indicate the presence or absence of 20 symptoms in the prior month by answering yes (scored 1) or no (scored 0) to each item. Items are summed to provide a total score (range 0–20), with higher scores indicating greater symptomatology. The SRQ-20 has been translated and validated for use in Uganda and demonstrated good internal consistency (Cronbach’s α = 0.84) and moderate test–retest reliability (κ = 0.48), and a cutoff score of ≥6 identified current depression with a sensitivity of 84% and specificity of 93% (66). The SRQ-20 was administered to caregivers to assess psychological distress and demonstrated good internal consistency (Cronbach’s α = 0.83).

##### HIV Stigma

To assess for HIV-related stigma, adolescents were asked five questions (yes/no) pertaining to stigma experienced in the prior year. The questions asked about i) being teased at home because of HIV status, ii) being teased at school/work because of HIV status, iii) being discriminated at home because of HIV status, iv) being discriminated at school/work because of HIV status, and v) having lost friends because of HIV status. Adolescents responding “yes” to any of the questions were regarded as having experienced HIV stigma in the prior year (yes/no).

#### Clinical Correlates

*Child/adolescent characteristics*: Gender (male/female), age (continuous in years), and whether the child missed any days of school in the last term (yes/no).

*Household characteristics*: Study site (rural/urban), who the child lives with (two parents, single parent, grandparents, other), food security (based on whether the household had enough food to eat in the prior month, yes/no), and socioeconomic index based on common household items (0–2, 3–4, 5–6, 7–9 items) constructed for use in Uganda (67) (including the following items: electricity, a car, a motorcycle, a bicycle, a radio, a telephone, a refrigerator, a cupboard, and a flask).

*HIV characteristics*: Whether the child was born with HIV (yes/no), nadir CD4 cell count (<200, 200–349, 350–499, 500+ cells/mm^3^), current CD4 cell count (< 200, 200–349, 350–499, 500+ cells/mm^3^), reported WHO HIV clinical stage (stage 1, stage 2, stage 3, stage 4), currently on antiretroviral treatment (ART, yes/no), possible virological treatment failure (current viral load > 1,000 copies/ml, yes/no), whether the adolescent missed any ARV doses in the past 3 days (yes/no), and whether HIV status has been disclosed to the child (yes/no).

*Caregiver characteristics*: Gender (male/female), age (continuous in years), caregiver status (mother, father, grandparent, other), employment (yes/no), HLOE (no formal education, primary education, secondary education, tertiary education), marital status (cohabiting, widowed, separated, single), caregiver also caring for other children (yes/no), caregiver HIV positive (yes/no), and caregiver health status (poor or average, good or very good).

*EBPs*: Rates of individual EBPs (as listed in **Table 1**) based on symptom cutoff scores on diagnostic measures are reported. Emotional problems (EPs) were considered present if the child or adolescent fulfilled symptom criteria for at least one EP. Behavioral problems (BPs) were considered present if the adolescent fulfilled symptom criteria for at least one BP. Similar to other studies that have used diagnostic tools (68, 69), we calculated total severity scores for EPs and BPs by adding the severity scores for the individual disorders (as presented in **Table 1**). With this approach, each symptom assessed would contribute equally to overall severity; however, total severity scores could be more heavily influenced by disorders with a greater number of symptoms. We, therefore, also calculated averaged total severity scores by dividing the severity score for each disorder by the number of symptoms assessed, which we used to conduct sensitivity analysis. By using averaged severity scores, each disorder would contribute equally to overall severity; however, disorders that were assessed with limited screening questions would be weighted equivalently to disorders that had been assessed in full.

### Statistical Analysis

The sample size of 1339 was based on an estimated prevalence of at least one psychiatric disorder of 25% with a precision of around 2.5%. Analyses were conducted separately for the child and adolescent samples as they completed different self-report questionnaires; “adolescents” aged 12–17 years completed the YI-4 and “children” aged 8–11 years completed the CI-4. Descriptive data include rates (%) of self-rated and caregiver-rated EBPs and sociodemographic and clinical variables (as specified above). We compared the rates reported by the caregivers and CA-HIV by conducting chi-square or Fisher’s exact tests as indicated.

Agreement between caregiver- and youth self-rated disorders was evaluated with the kappa statistic based on symptom cutoff scores (i.e., categorical) and Pearson correlation coefficients for severity scores (i.e., dimensional). To measure the discrepancy between caregivers and CA-HIV, we used a recommended approach by calculating standardized difference scores (69). We transformed each informant’s total EP and total BP severity scores into *z* scores. We subtracted the *z* score obtained for the CA-HIV from the *z* score obtained for the caregivers to obtain the standardized difference (discrepancy) score for EPs and BPs. Positive scores indicate caregivers rating problems as more severe and negative scores indicate CA-HIV rating problems as more severe. We repeated the same process using averaged severity scores to compute the discrepancy scores, which were used in sensitivity analyses.

To identify clinical and sociodemographic factors that were associated with discrepancy between caregivers and CA-HIV on EPs or BPs, *t* tests and analyses of variance (ANOVAs) were conducted for categorical variables and Pearson correlation coefficients for continuous variables. Variables with a *p* value of less than 0.1 were entered into multiple linear regression models to assess factors that each best predicted the discrepancy between caregivers and CA-HIV of EPs and BPs. CA-HIV age and gender were included in each of the models, even if their *p* values were not significant on univariate analyses. To assess for the effects of HIV stigma and caregiver psychological distress on discrepancy scores, we added the variables “experienced HIV stigma” and “SRQ-20 total score” as an additional step to the final model. We conducted sensitivity analyses by repeating the regression models, but with discrepancy scores based on averaged severity scores as the dependent variables. We did not correct for multiple comparisons due to the exploratory nature of the analyses. Analyses were conducted using SPSS version 25 software package (SPSS Inc., Chicago, IL), and all tests were two-tailed with the alpha (α) set at 0.05.

## Results

### Participants and Descriptive Data

The overall sample of 1,339 included 351 children under the age of 8 years who did not qualify for inclusion in this study, as they were too young to complete the CI-4. We excluded one adolescent aged 18 years and three CA-HIV for whom age data were missing. We excluded a further 18 children who had not completed the CI-4 and four adolescents who had not completed the YI-4. Our final sample included 493 children who had completed the CI-4 and 469 adolescents who had completed the YI-4. CASI-5 data were missing for 28 (5.9%) adolescents, and five (1.0%) children were excluded from the analysis as the caregivers who completed the CASI-5 were not above the age of 17 years. Factors associated with adolescents who were excluded (*n* = 33, 7.0%) were older age [*t*(472) = 3.1, *p* = 0.002], urban study site [χ²(1) = 25.9, *p* < 0.001], a higher socioeconomic index [χ²(3) = 8.0, *p* = 0.047], caregivers interviewed not the parents or grandparents [χ²(3) = 24.7, *p* < 0.001], and caregivers being employed [χ²(1) = 5.3, *p* = 0.021]. Factors associated with children who were excluded (*n* = 23, 4.5%) were urban study site [χ²(1) = 10.7, *p* = 0.001], WHO clinical stage 1 or 2 [χ²(3) = 8.0, *p* = 0.046], caregiver younger [*t*(472) = −2.5, *p* = 0.012], and caregiver not HIV positive [χ²(1) = 4.1, *p* = 0.042].

The mean age of CA-HIV was 11.9 (SD = 2.6) years, and 52.9% of CA-HIV were female. The majority of CA-HIV (94.2%) were perinatally infected and were receiving ART (95.4%). Eighty-seven (19.7%) of the adolescents reported experiencing HIV-related stigma in the prior year, with 31 (7.0%) answering “yes” to two or more questions. Of the caregivers assessed, 177 (19.1%) scored ≥6 on the SRQ-20 (the threshold indicating possible depression in the Ugandan validation study), and the median score on the SRQ-20 was 4.0 (IQR 0.0; 4.0). For detailed descriptive data, see **Tables 3** and **4**.

### Rates of EBPs (**Table 2**)

**Table 2:** Rates of caregiver and self-rated emotional and behavioral problems.

Problems assessed	Caregiver	Adolescent	χ²	*p* value	Caregiver	Child	χ²	*p* value
	*n* (%)	*n* (%)			*n* (%)	*n* (%)		
Total sample	441 (94.0)	469 (100)			488 (99.0)	493 (100)		
Total problems	273 (58.2)	166 (35.4)	63.97	<0.001*				
Total behavioral problems	43 (9.2)	68 (14.5)	4.79	0.029*				
ADHD	19 (4.3)	17 (3.6)	0.28	0.597				
ODD	15 (3.4)	18 (3.8)	0.12	0.725				
CD	25 (5.7)	44 (9.4)	4.47	0.034*				
SUD	2 (0.5)	18 (3.8)	12.11	<0.001*				
Total emotional problems	261 (55.7)	135 (28.8)	85.45	<0.001*	270 (54.8)	182 (36.9)	33.46	<0.001*
GAD	7 (1.6)	14 (3.0)	1.97	0.160	17 (3.4)	29 (5.9)	3.16	0.076
Specific phobia	189 (42.9)	77 (16.4)	76.80	<0.001*	223 (45.2)	124 (25.2)	45.28	<0.001*
Panic disorder	92 (20.9)	38 (8.1)	30.22	<0.001*				
SAD	8 (1.8)	49 (10.4)	28.86	<0.001*	14 (2.8)	77 (15.6)	47.37	<0.001
Separation AD	25 (5.7)	10 (2.1)	7.69	0.006*	27 (54.8)	41 (8.3)	2.95	0.086
MDE	11 (2.5)	4 (0.9)	3.78	0.068	5 (1.0)	2 (0.4)	1.33	0.285
PDD	19 (4.3)	24 (5.1)	0.33	0.565	9 (1.8)	14 (2.8)	1.06	0.303
PTSD	85 (19.1)	16 (3.4)	57.96	<0.001*	57 (11.6)	7 (1.4)	42.34	<0.001*
SSD	61 (13.8)	19 (4.1)	27.12	<0.001*	58 (11.8)	21 (4.3)	19.26	<0.001*

*Significance set at p < 0.05.

AD, anxiety disorder; ADHD, attention deficit hyperactivity disorder; CD, conduct disorder; GAD, generalized anxiety disorder; MDE, major depressive episode; ODD, oppositional defiant disorder; PDD, persistent depressive disorder; PTSD, posttraumatic stress disorder; SAD, social anxiety disorder; SSD, somatic symptom disorder; SUD, substance use disorder.

#### Rates of Behavioral Problems

##### Adolescents

Based on symptom count cutoff scores, caregiver- and self- reported BPs were present among 9.2% and 14.5% of adolescents, respectively. Adolescents self-reported a mean number of 0.21 (SD = 0.58) BPs, and of those reporting BPs, 30.9% reported having at least two BPs. Adolescents self-reported more BPs than their caregivers [χ²(1) = 4.8, *p* = 0.029], particularly conduct disorder [χ²(1) = 4.5, *p* = 0.034] and substance use problems [χ²(1) = 12.1, *p* < 0.001].

#### Rates of Emotional Problems

##### Adolescents

Caregivers reported that 55.7% of adolescents met symptom count criteria for an EP compared with 28.8% according to adolescent self-report. The mean number of self-reported EPs was 0.54 (SD = 1.12), and of those adolescents reporting EPs, 43.7% met criteria for at least two, and 21.5% met criteria for at least three EPs. Caregivers reported more EPs than adolescents [χ²(1) = 85.5, *p* < 0.001], specifically higher rates of specific phobia [χ²(1) = 76.8, *p* < 0.001], panic attacks [χ²(1) = 30.2, *p* < 0.001], posttraumatic stress disorder (PTSD) [χ²(1) = 58.0, *p* < 0.001], somatic symptoms [χ²(1) = 27.1, *p* < 0.001], and separation anxiety disorder [χ²(1) = 7.7, *p* = 0.006]. Adolescents self-reported higher rates of social anxiety disorder [SAD, χ²(1) = 28.9, *p* < 0.001].

##### Children

Caregivers indicated that 54.8% of the children met criteria for an EP whereas 36.9% of the children self-reported EPs. The mean number of self-reported EPs was 0.64 (1.07), and of the children self-reporting EPs, 42.9% reported at least two EPs and 18.1% reported three or more. Caregivers reported more EPs than children [χ²(1) = 33.5, *p* < 0.001], specifically specific phobia [χ²(1) = 45.3, *p* < 0.001], PTSD [χ²(1) = 42.3, *p* < 0.001], and somatic symptoms [χ²(1) = 19.3, *p* < 0.001]. Children self-reported higher rates of SAD [χ²(1) = 47.4, *p* < 0.001].

#### Co-Occurrence

Comorbidity of EPs and BPs was also noted, with 41 (8.7%) adolescents and 31 (7.0%) caregivers reporting both EPs and BPs. The number of EPs reported was significantly correlated with the number of BPs reported by both caregivers (*r* *_s_* = 0.14, *p* = 0.004) and adolescents (*r* *_s_* = 0.29, *p* < 0.001).

### Caregiver and Self-Report Agreement (**Table 3**)

**Table 3:** Agreement between caregiver-rated and CA-HIV self-rated emotional and behavioral problems.

	Caregiver–adolescent agreement				Caregiver–child agreement			
	Kappa	*p* value	Correlation	*p* value	Kappa	*p* value	Correlation	*p* value
Behavioral problems	0.122	0.009*	0.252	<0.001*				
ADHD	0.073	0.123	0.286	<0.001*				
ODD	0.092	0.052	0.210	<0.001*				
CD	0.090	0.050	0.160	0.001*				
SUD	−0.008	0.770	−0.012	0.802				
Emotional problems	0.062	0.109	0.197	<0.001*	0.151	<0.001*	0.190	<0.001*
GAD	0.087	0.058	0.250	<0.001*	0.001	0.979	0.129	0.004*
Specific phobia	0.006	0.876	0.162	0.001*	0.067	0.103	0.178	<0.001*
Panic disorder	−0.009	0.827	0.067	0.162				
SAD	0.085	0.010*	0.152	0.001*	0.042	0.174	0.081	0.075
Separation AD	0.026	0.549	0.138	0.004*	0.055	0.216	0.108	0.017*
MDE	−0.013	0.748	0.209	<0.001*	−0.006	0.885	0.182	<0.001*
PDD	0.047	0.318	0.165	<0.001*	−0.023	0.603	0.175	<0.001*
PTSD	0.037	0.211	0.080	0.092	0.038	0.161	0.090	0.047*
SSD	0.037	0.351	0.169	<0.001*	0.122	0.002*	0.203	<0.001*

*Significance set at p < 0.05.

AD, anxiety disorder; ADHD, attention deficit hyperactivity disorder; CD, conduct disorder; GAD, generalized anxiety disorder; MDE, major depressive episode; ODD, oppositional defiant disorder; PDD, persistent depressive disorder; PTSD, posttraumatic stress disorder; SAD, social anxiety disorder; SSD, somatic symptom disorder; SUD, substance use disorder.

Informant agreement was poor (κ between −0.023 and 0.122), with a maximum kappa of 0.122 (*p* = 0.002) for somatic symptom presence. There was also only modest agreement based on severity scores (*r* between −0.012 and 0.286).

### Factors Associated With Caregiver and CA-HIV Discrepancy

Discrepancy scores were all positive, indicating that overall caregivers rated increased severity of EBPs than CA-HIV. The discrepancy scores between EPs and BPs in adolescents were also significantly correlated (*r* = 0.66, *p* < 0.001). The association between caregiver and CA-HIV discrepancy and sociodemographic and clinical factors based on univariate analyses are reported in **Tables 4** and **5** for adolescents and children, respectively.

**Table 4:** Association of demographic and clinical variables to the standardized difference score for emotional and behavioral problems between caregivers and adolescents.

		Emotional problems			Behavioral problems		
Variable	Total sample	Std difference score	Test statistic	*p* value	Std difference score	Test statistic	*p* value
		M (SD)			M (SD)		
Total sample *n* (%)	441 (100)	0.00 (1.27)			0.00 (1.22)		
Child/adolescent characteristics							
Gender *n* (%)			*t* (439) = −1.38	0.169		*t* (439) = −1.35	0.179
Female	230 (52.2)	−0.08 (1.21)			−0.08 (1.15)		
Male	211 (47.8)	0.09 (1.32)			0.08 (1.30)		
Age M (SD)	13.88 (1.44)		*r* = −0.13	0.009*		*r* = −0.09	0.072
Missed any school *n* (%)			*t* (401) = −0.64	0.521		*t* (401) = 0.12	0.906
No	170 (38.5)	−0.05 (1.25)			0.02 (1.20)		
Yes	233 (57.8)	0.03 (1.29)			0.00 (1.21)		
Experienced HIV stigma *n* (%)			*t* (439) = −2.19	0.029*		*t* (439) = −0.86	0.391
No	354 (80.3)	0.06 (1.3)			0.03 (1.2)		
Yes	87 (19.7)	−0.27 (1.3)			−0.10 (1.3)		
Household characteristics							
Study site *n* (%)			*t* (439) = −1.76	0.079		*t* (439) = 0.41	0.679
Rural	215 (48.8)	−0.11 (1.16)			0.03 (1.21)		
Urban	226 (51.2)	0.10 (1.35)			−0.02 (1.23)		
Lives with *n* (%)			*F* (3, 437) = 0.21	0.889		*F* (3, 437) = 1.86	0.136
Two parents	106 (24.0)	0.04 (1.26)			−0.03 (1.04)		
Single parent	143 (32.4)	−0.07 (1.29)			−0.15 (1.20)		
Grandparents	87 (19.7)	0.05 (1.08)			0.23 (1.19)		
Other	105 (23.8)	0.02 (1.40)			0.04 (1.42)		
Food security *n* (%)			*t* (436) = 0.34	0.731		*t* (436) = −1.58	0.116
No	94 (21.3)	0.04 (1.37)			−1.76 (1.38)		
Yes	344 (78.5)	−0.01 (1.24)			0.05 (1.18)		
Socioeconomic index *n* (%)			*F* (3, 430) = 0.85	0.470		*F* (3, 430) = 0.33	0.804
0–2	37 (8.5)	0.15 (1.02)			0.11 (1.13)		
3–4	144 (33.2)	0.00 (1.23)			0.03 (1.22)		
5–6	178 (41.0)	−0.10 (1.40)			−0.01 (1.30)		
7–9	75 (17.3)	0.14 (1.13)			−0.11 (1.13)		
HIV related							
Born with HIV *n* (%)			*t* (422) = 0.26	0.792		*t* (13.6) < 0.01	1.000
No	14 (3.2)	0.09 (1.46)			−0.01 (1.56)		
Yes	410 (96.7)	0.00 (1.27)			−0.01 (1.22)		
Nadir CD4 count *n* (%)			*F* (3, 399) = 0.98	0.402		*F* (3, 399) = 0.67	0.572
<200	81 (20.1)	−0.06 (1.35)			0.07 (1.47)		
200–349	73 (18.1)	0.08 (1.24)			0.07 (1.25)		
350–499	71 (17.6)	−0.26 (1.10)			−0.16 (0.91)		
500+ cells/mm^3^	178 (44.2)	−0.02 (1.27)			−0.08 (1.22)		
Current CD4 count *n* (%)			*F* (3, 430) = 1.02	0.382		*F* (3, 430) = 1.77	0.152
<200	26 (6.0)	0.15 (1.47)			0.24 (1.16)		
200–349	49 (11.3)	0.05 (1.55)			0.24 (1.38)		
350–499	69 (15.9)	0.22 (1.10)			0.13 (0.97)		
500+ cells/mm^3^	290 (66.8)	−0.06 (1.24)			−0.09 (1.24)		
WHO HIV clinical stage *n* (%)			*F* (3, 437) = 3.83	0.010*		*F* (3, 437) = 1.52	0.207
Stage 1	57 (12.9)	−0.12 (1.47)			−0.05 (1.46)		
Stage 2	220 (49.9)	0.05 (1.28)			0.04 (1.17)		
Stage 3	155 (35.1)	−0.11 (1.08)			−0.08 (1.21)		
Stage 4	9 (2.0)	1.29 (1.86)			0.77 (0.76)		
On ART *n* (%)			*t* (439) = 0.22	0.824		*t* (439) = 0.60	0.551
No	25 (5.7)	0.05 (1.58)			0.14 (1.55)		
Yes	416 (94.3)	−0.03 (1.25)			−0.01 (1.20)		
Viral load > 1000 copies/ml^a^			*t* (254) = −0.43	0.667		*t* (254) = −0.29	0.772
No	176 (39.9)	−0.02 (1.30)			−0.03 (1.32)		
Yes	80 (31.3)	0.05 (1.25)			0.02 (1.06)		
Missed any ARV doses *n* (%)			*t* (427) = −1.06	0.288		*t* (427) = −0.34	0.737
No	396 (89.8)	−0.21 (1.17)			−0.06 (1.35)		
Yes	33 (7.7)	0.03 (1.27)			0.01 (1.20)		
Caregiver characteristics							
Gender *n* (%)			*t* (439) = 0.02	0.986		*t* (439) = −1.67	0.097
Female	355 (80.5)	0.00 (1.28)			−0.05 (1.21)		
Male	86 (19.5)	−0.00 (1.20)			0.20 (1.26)		
Age M (SD)	41.58 (11.95)		*r* = −0.10	0.036*		*r* = −0.42	0.381
Caregiver interviewed *n* (%)			*F* (3, 437) = 2.06	0.105		*F* (3, 437) = 1.23	0.297
Mother	191 (43.3)	−0.03 (1.31)			−0.10 (1.20)		
Father	46 (10.4)	−0.22 (1.03)			−0.05 (0.91)		
Grandparent	62 (14.1)	−0.19 (1.01)			−0.02 (1.07)		
Other	142 (32.2)	1.94 (1.36)			0.16 (1.39)		
Employed *n* (%)			*t* (74.8) = −1.57	0.121		*t* (439) = −2.10	0.036*
No	62 (14.1)	−0.27 (1.50)			−0.30 (1.49)		
Yes	379 (85.9)	0.04 (1.22)			0.05 (1.17)		
HLOE *n* (%)			*F* (3, 435) = 5.14	0.002*		*F* (3, 435) = 1.74	0.159
No formal education	42 (9.6)	−0.34 (0.97)			0.01 (1.17)		
Primary education	196 (44.6)	−0.13 (1.13)			−0.08 (1.12)		
Secondary education	140 (31.9)	0.06 (1.37)			−0.03 (1.25)		
Tertiary education	61 (13.9)	0.50 (1.50)			0.32 (1.46)		
Marital status *n* (%)			*F* (3, 437) = 1.21	0.304		*F* (3, 437) = 0.82	0.482
Cohabiting	229 (51.9)	−0.03 (1.24)			0.02 (1.26)		
Widowed	109 (24.7)	−0.05 (1.35)			−0.06 (1.15)		
Separated	47 (10.7)	−0.10 (1.20)			−0.16 (1.10)		
Single	56 (12.7)	0.30 (1.28)			0.19 (1.33)		
Carer of other children *n* (%)			*t* (439) = −1.99	0.047*		*t* (439) = −1.75	0.080
No	82 (18.6)	−0.25 (1.10)			−0.21 (1.10)		
Yes	359 (81.4)	0.06 (1.30)			0.05 (1.25)		
Caregiver HIV positive *n* (%)			*t* (435) = 0.63	0.530		*t* (435) = 1.97	0.049*
No	196 (44.4)	0.05 (1.28)			0.13 (1.31)		
Yes	241 (55.1)	−0.02 (1.24)			−0.10 (1.15)		
Caregiver health status *n* (%)			*t* (439) = 1.55	0.123		*t* (439) = 0.62	0.535
Poor or average	195 (44.2)	0.10 (1.40)			0.04 (1.25)		
Good or very good	246 (55.8)	−0.08 (1.14)			−0.03 (1.20)		
SRQ-20 score ≥ 6 *n* (%)			*t* (87.2) = −2.38	0.020*		*t* (84.1) = −1.18	0.243
No	370 (83.9)	−0.07 (1.2)			−0.04 (1.1)		
Yes	71 (16.1)	0.38 (1.5)			0.20 (1.6)		
SRQ-20 total score M (SD)	2.78 (3.38)		*t* = 0.17	<0.001*		*t* = 0.10	0.045*

a Viral load missing for 185 (42.0%) of adolescents.

*Significance set at p < 0.05.

ART, antiretroviral treatment; BPs, behavioral problems; EPs, emotional problems; HLOE, highest level of education; SRQ-20, Self-Reporting Questionnaire.

**Table 5:** Association of demographic and clinical variables to the standardized difference score for emotional problems between caregivers and children.

Variable	Total sample	Std difference score	Test statistic	*p* value
		M (SD)		
Total sample *n* (%)	488 (100)	0.00 (1.27)		
Child/adolescent characteristics				
Gender *n* (%)				
Female			*t* (486) = 0.11	0.913
Male	256 (52.5)	0.01 (1.29)		
	232 (47.5)	0.00 (1.26)		
Age M (SD)	9.42 (1.09)		*r* = −0.12	0.011*
Missed any school *n* (%)			*t* (472) = 0.21	0.838
No	187 (38.3)	−0.01 (1.11)		
Yes	287 (60.5)	−0.03 (1.33)		
Household characteristics				
Study site *n* (%)			*t* (486) = −1.92	0.056
Rural	255 (52.3)	−0.11 (1.32)		
Urban	233 (47.7)	0.12 (1.22)		
Lives with *n* (%)			*F* (3, 484) = 1.90	0.318
Two parents	144 (29.6)	0.07 (1.39)		
Single parent	191 (39.2)	0.07 (1.23)		
Grandparents	91 (18.7)	−0.21 (1.31)		
Other	61 (12.5)	−0.05 (1.03)		
Food security *n* (%)			*t* (485) = 1.53	0.126
No	101 (20.7)	0.17 (1.36)		
Yes	386 (79.3)	−0.04 (1.25)		
Socioeconomic index *n* (%)			*F* (3, 480) = 0.15	0.927
0–2	83 (17.1)	0.05 (1.30)		
3–4	174 (36.0)	0.02 (1.33)		
5–6	166 (34.3)	0.00 (1.13)		
7–9	61 (12.6)	−0.09 (1.40)		
HIV related				
Born with HIV *n* (%)			*t* (474) = 0.69	0.492
No	10 (2.0)	0.27 (1.44)		
Yes	466 (97.9)	−0.01 (1.26)		
Nadir CD4 count *n* (%)			*F* (3, 449) = 0.32	0.812
<200	57 (12.6)	0.09 (1.48)		
200–349	51 (11.3)	−0.06 (1.37)		
350–499	47 (10.4)	−0.12 (1.60)		
500+ cells/mm3	298 (65.8)	0.05 (1.19)		
Current CD4 count *n* (%)			*F* (3, 482) = 0.92	0.433
<200	26 (5.3)	−0.26 (1.23)		
200–349	14 (2.8)	−0.31 (0.93)		
350–499	38 (7.8)	0.17 (1.44)		
500+ cells/mm3	408 (84.0)	0.02 (1.26)		
WHO HIV clinical stage *n* (%)			*F* (3, 484) = 2.84	0.037*
Stage 1	74 (15.2)	0.12 (1.14)		
Stage 2	275 (56.4)	0.08 (1.22)		
Stage 3	131 (26.8)	−0.27 (1.41)		
Stage 4	8 (1.6)	0.44 (1.57)		
On ART *n* (%)			*t* (466) = 0.54	0.587
No	469 (96.1)	0.16 (0.97)		
Yes	19 (3.9)	−0.01 (1.28)		
Viral load > 1000 copies/ml			*t* (466) = 0.24	0.808
No	116 (24.8)	0.02 (1.26)		
Yes	352 (72.1)	−0.01 (1.33)		
Disclosed HIV status *n* (%)			*t* (486) = 0.18	0.986
No	274 (56.1)	0.00 (1.31)		
Yes	214 (43.9)	−0.00 (1.23)		
Caregiver characteristics				
Gender *n* (%)			*t* (486) = −0.01	0.996
Female	413 (84.6)	−0.00 (1.29)		
Male	75 (15.4)	0.00 (1.21)		
Age M (SD)	39.6 (11.7)		*r* = −0.12	0.011*
Caregiver interviewed *n* (%)			*F* (3, 484) = 1.97	0.118
Mother	270 (55.3)	0.09 (1.29)		
Father	49 (10.0)	−0.06 (1.32)		
Grandparent	68 (13.9)	−0.33 (1.33)		
Other	101 (20.7)	0.02 (1.14)		
Employed *n* (%)			*t* (486) = 0.48	0.633
No	73 (15.0)	0.66 (1.42)		
Yes	415 (85.0)	−0.01 (1.25)		
HLOE *n* (%)			*F* (3, 484) = 4.37	0.005*
No formal education	47 (9.6)	−0.46 (1.28)		
Primary education	249 (51.0)	−0.08 (1.21)		
Secondary education	153 (31.4)	0.20 (1.35)		
Tertiary education	39 (8.0)	0.28 (1.17)		
Marital status *n* (%)			*F* (3, 484) = 0.38	0.770
Cohabiting	250 (51.2)	0.05 (1.27)		
Widowed	83 (17.0)	−0.06 (1.43)		
Separated	91 (18.6)	−0.09 (1.13)		
Single	64 (13.1)	0.00 (1.27)		
Carer of other children *n* (%)			*t* (484) = −0.75	0.453
No	67 (13.7)	−0.11 (1.30)		
Yes	419 (86.2)	0.02 (1.27)		
Caregiver HIV positive *n* (%)			*t* (474) = −1.62	0.105
No	134 (27.5)	−0.15 (1.14)		
Yes	342 (71.8)	0.06 (1.30)		
Caregiver health status *n* (%)			*t* (485) = 2.01	0.045*
Poor or average	247 (50.7)	0.11 (1.33)		
Good or very good	240 (49.3)	−0.12 (1.20)		
SRQ-20 score ≥ 6 *n* (%)			*t* (140.5) = −3.74	<0.001*
No	382 (78.3)	−0.13 (1.2)		
Yes	106 (21.7)	0.47 (1.5)		
SRQ-20 total score M (SD)	3.25 (3.51)		*r* = 0.22	<0.001*

*Significance set at p < 0.05.

ART, antiretroviral treatment; HLOE, highest level of education; SRQ-20, Self-Reporting Questionnaire.

#### Adolescent Behavior Problems

Discrepancy scores for BPs were significantly different by caregiver employment status [*t*(439) = −2.10, *p* = 0.036] and caregiver HIV status [*t*(435) = 1.97, *p* = 0.049]. These variables were added to the linear regression model along with caregiver caring for other children [*t*(439) = −1.75, *p* = 0.080], caregiver gender [*t*(439) = −1.67, *p* = 0.097], adolescent gender [*t*(439) = −1.35, *p* = 0.179], and adolescent age (*r* = −0.09, *p* = 0.072) (**Table 6**). Discrepancy on BP scores were associated with adolescent age (*B* = −0.09, 95% CI −0.17; −0.01, *p* = 0.037), caregiver employment status (*B* = 0.33, 95% CI 0.00; 0.67, *p* = 0.049), and caregiver HIV status (*B* = −0.26, 95% CI −0.50; −0.02, *p* = 0.037). Discrepancy scores decreased with advancing adolescent age and caregivers rated BPs as less severe than adolescents if caregivers were HIV positive and unemployed.

**Table 6:** Linear regression of factors associated with discrepancy in the presence of behavioral problems between caregivers and adolescents.

Variable	*B* (95% CI)	**β**	*p* value
Model 1			
Constant	0.78 (−0.45; 2.01)		0.214
Adolescent age	−0.09 (−0.17; −0.01)	−0.10	0.037*
Adolescent male	0.11 (−0.12; 0.34)	0.04	0.356
Caregiver male	0.18 (−0.12; 0.47)	0.06	0.240
Carer other children	0.24 (−0.05; 0.53)	0.08	0.107
Caregiver employed	0.33 (0.00; 0.67)	0.10	0.049*
Caregiver HIV positive	−0.26 (−0.50; −0.02)	−0.10	0.032*
Model 2			
Constant	0.61 (−0.63; 1.84)		0.334
Adolescent age	−0.08 (−0.16; −0.00)	−0.10	0.049*
Adolescent male	0.13 (−0.10; 0.36)	0.05	0.264
Caregiver male	0.20 (−0.10; 0.49)	0.06	0.187
Carer other children	0.25 (−0.05; 0.54)	0.08	0.099
Caregiver employed	0.33 (0.00; 0.66)	0.10	0.049*
Caregiver HIV positive	−0.29 (−0.53; −0.06)	−0.12	0.014*
Experienced HIV stigma	−0.11 (−0.39; 0.18)	−0.03	0.463
SRQ-20 total score	0.04 (0.01; 0.08)	0.12	0.011*

Model 1: F (6, 403) = 3.17 (p = 0.005*), R² = 0.042.

Model 2: F (8, 428) = 3.28 (p = 0.001*), R² = 0.058; R² change = 0.015 (p = 0.031*).

*Significance set at p < 0.05.

B, the unstandardized regression coefficient or beta; β, the standardized beta; SRQ-20, Self-Reporting Questionnaire.

Discrepancy scores were significantly associated with caregiver SRQ-20 scores (*r* = 0.10, *p* = 0.045), but not with HIV stigma experienced by adolescents [*t*(439) = −0.86, *p* = 0.391]. When these variables were added to the model, the model was significantly improved (*R*² change = 0.015, *p* = 0.031) and discrepancy scores were significantly associated with caregiver SRQ-20 scores (*B* = 0.04, 95% CI 0.01; 0.08, *p* = 0.011), such that caregivers with higher levels of psychological distress rated BPs as being more severe than adolescents self-rated.

#### Adolescent Emotional Problems

Discrepancy scores for EPs were significantly associated with adolescent age (*r* = −0.13, *p* = 0.009), CA-HIV WHO HIV clinical stage [*F*(3, 437) = 3.83, *p =* 0.010], caregiver age (*r* = −0.10, *p* = 0.036), caregiver HLOE [*F*(3, 435) = 5.14, *p* = 0.002], and caregiver caring for other children [*t*(439) = −1.99, *p* = 0.047]. These variables were added to the linear regression model along with adolescent gender [*t*(439) = −1.38, *p* = 0.169] and study site [*t*(439) = −1.76, *p* = 0.079] (**Table 7**). Discrepancy in EPs was significantly associated with WHO HIV stage 4 (*B* = 1.47, 95% CI 0.59; 2.34, *p* = 0.001), caregivers caring for other children (*B* = 0.32, 95% CI 0.02; 0.62, *p* = 0.038), and a tertiary HLOE in caregivers (*B* = 0.77, 95% CI 0.26; 1.27, *p* = 0.003). Caregivers rated EPs as being more severe than adolescents if the adolescents had WHO HIV stage 4 compared to stage 1, if the caregiver had a tertiary HLOE as compared to no formal education, and if the caregiver was also caring for other children.

**Table 7:** Linear regression of factors associated with discrepancy in the presence of emotional problems between caregivers and adolescents.

Variable	*B* (95% CI)	**β**	*p* value
Model 1			
Constant	0.60 (−0.77; 1.97)		0.393
Adolescent age	−0.09 (−0.17; 0.00)	−0.10	0.054
Adolescent male	0.10 (−0.14; 0.34)	0.04	0.423
Urban environment	0.03 (−0.23; 0.29)	0.01	0.827
WHO HIV stage 2^a^	0.31 (−0.08; 0.70)	0.12	0.117
WHO HIV stage 3^a^	0.20 (−0.20; 0.60)	0.08	0.325
WHO HIV stage 4^a^	1.47 (0.59; 2.35)	0.17	0.001*
Caregiver age	−0.01 (−0.02; 0.00)	−0.07	0.170
Carer other children	0.32 (0.02; 0.62)	0.10	0.038*
Caregiver HLOE primary^b^	0.16 (−026; 0.58)	0.06	0.447
Caregiver HLOE secondary^b^	0.29 (−0.15; 0.72)	0.10	0.203
Caregiver HLOE tertiary^b^	0.77 (0.26; 1.27)	0.21	0.003*
Model 2			
Constant	0.41 (−0.95; 1.76)		0.555
Adolescent age	−0.08 (−0.16; 0.00)	−0.10	0.057
Adolescent male	0.13 (−0.14; 0.34)	0.05	0.278
Urban environment	−0.11 (−0.37; 0.15)	−0.04	0.419
WHO HIV stage 2^a^	0.35 (−0.03; 0.73)	0.14	0.068
WHO HIV stage 3^a^	0.20 (−0.19; 0.60)	0.08	0.305
WHO HIV stage 4^a^	1.50 (0.64; 2.37)	0.17	0.001*
Caregiver age	−0.01 (−0.02; 0.00)	−0.06	0.186
Carer other children	0.30 (−0.03; 0.73)	0.09	0.057
Caregiver HLOE primary^b^	0.16 (−0.18; 0.64)	0.09	0.276
Caregiver HLOE secondary^b^	0.29 (−0.05; 0.81)	0.14	0.086
Caregiver HLOE tertiary^b^	0.77 (0.32; 1.31)	0.22	0.001*
Experienced HIV stigma	−0.37 (−0.67; −0.08)	−0.12	0.012*
SRQ-20 total score	0.07 (0.03; 0.10)	0.18	<0.001*

Model 1: F (11, 423) = 3.69 (p < 0.001*), R² = 0.087. 11

Model 2: F (13, 421) = 4.79 (p < 0.001*), R² = 0.129; R² change = 0.041 (p < 0.001*).

*Significance set at p < 0.05

a WHO stage 1 was the WHO HIV stage category against which other WHO stage categories were analyzed.

b No formal education was the education category against which other education categories were analyzed.

B, the unstandardized regression coefficient or beta; β, the standardized beta; HLOE, highest level of education; SRQ-20, Self-Reporting Questionnaire.

Discrepancy scores were significantly associated with caregiver SRQ-20 scores (*r* = 0.17, *p* < 0.001) and with HIV stigma experienced by adolescent [*t*(439) = −2.19, *p* = 0.029]. The model was significantly improved (*R*² change = 0.041, *p* < 0.001) when these variables were added and discrepancy scores were significantly associated with HIV stigma experienced by adolescents (*B* = −0.37, 95% CI −0.67; −0.08, *p* = 0.012) and with caregiver SRQ-20 scores (*B* = 0.07, 95% CI 0.03; 0.10, *p* = 0.011). Caring for other children was no longer significantly associated with discrepancy scores (*B* = 0.30, 95% CI −0.03; 0.73, *p* = 0.057). Adolescents who had experienced stigma in the prior year rated EPs as more severe than caregivers and caregivers with increased psychological distress rated EPs more severe than adolescents self-rated.

To assess for possible effects related to the context in which adolescents had experienced HIV-related stigma, we performed *post hoc* testing by repeating the final model, but dividing HIV stigma according to those who endorsed stigma at home only, outside the home only, or in both settings. Adolescents who experienced stigma at home only (*B* = −0.70, 95% CI −1.20; −0.20, *p* = 0.007), but not outside the home only (*B* = −0.31, 95% CI −0.68; 0.06, *p* = 0.097) or in both settings (*B* = 0.03, 95% CI −0.67; 0.74, p = 0.926), self-rated their EPs as significantly more severe than their caregivers rated, compared with adolescents who did not endorse experiencing HIV-related stigma.

#### Child Emotional Problems

Discrepancy scores for EPs were associated with child age (*r* = 0.10, *p* = 0.011), CA-HIV WHO HIV clinical stage [*F*(3, 484) = 2.84, *p* = 0.037], caregiver age (*r* = −0.12, *p* = 0.011), caregiver HLOE [*F*(3, 484) = 4.37, *p* = 0.005], and caregiver health status [*t*(485) = 2.01, *p* = 0.045]. These factors were added to the linear regression model along with study site [*t*(486) = −1.92, *p* = 0.056] and child gender [*t*(486) = 0.11, *p* = 0.913] (**Table 8**). Discrepancy in reporting EPs was significantly associated with child age (*B* = −0.16, 95% CI −0.26; −0.06, *p* = 0.003), caregiver age (*B* = −0.01, 95% CI −0.02; −0.00, *p* = 0.030), caregiver health status (*B* = −0.31, 95% CI −0.54; −0.08, *p* = 0.007), and caregivers having a primary (*B* = 0.43, 95% CI 0.04; 0.82, *p* = 0.031), secondary (*B* = 0.62, 95% CI 0.21; 1.04, *p* = 0.004), and tertiary HLOE (*B* = 0.80, 95% CI 0.25; 1.34, *p* = 0.004). Discrepancy scores decreased with advancing child and caregiver age. Caregivers rated EPs as more severe than children rated EPs if caregivers rated their own health status as “average” or “poor” as compared to “good” or “very good” or if caregivers had any level of education as compared to no formal education.

**Table 8:** Linear regression of factors associated with discrepancy on the presence of emotional problems between caregivers and children.

Variable	*B* (95% CI)	**β**	*p* value
Model 1			
Constant	1.58 (0.42; 2.74)		0.008*
Child age	−0.16 (−0.26; −0.06)	−0.14	0.003*
Child male	0.03 (−0.19; 0.25)	0.01	0.784
Urban environment	0.09 (−0.15; 0.34)	0.04	0.461
WHO HIV stage 2^a^	0.05 (−0.29; 0.39)	0.02	0.793
WHO HIV stage 3^a^	−0.27 (−0.64; 0.10)	−0.10	0.151
WHO HIV stage 4^a^	0.48 (−0.44; 1.39)	0.05	0.308
Caregiver age	−0.01 (−0.02; −0.00)	−0.10	0.030*
Caregiver health good	−0.31 (−0.54; −0.08)	−0.12	0.007*
Caregiver HLOE primary^b^	0.43 (0.04; 0.82)	0.17	0.031*
Caregiver HLOE secondary^b^	0.62 (0.21; 1.04)	0.23	0.004*
Caregiver HLOE tertiary^b^	0.80 (0.25; 1.34)	0.17	0.004*
Model 2			
Constant	1.16 (−0.00; 2.32)		0.050
Child age	−0.16 (−0.26; −0.06)	−0.13	0.002*
Child male	0.02 (−0.20; 0.24)	0.08	0.854
Urban environment	0.02 (−0.22; 0.27)	0.04	0.863
WHO HIV stage 2^a^	0.07 (−0.26; 0.41)	0.03	0.676
WHO HIV stage 3^a^	−0.23 (−0.60; 0.14)	−0.08	0.216
WHO HIV stage 4^a^	0.34 (−0.56; 1.25)	0.03	0.455
Caregiver age	−0.01 (−0.02; 0.00)	−0.08	0.079
Caregiver health good	−0.13 (−0.37; 0.11)	−0.05	0.273
Caregiver HLOE primary^b^	0.44 (0.05; 0.82)	0.17	0.025*
Caregiver HLOE secondary^b^	0.66 (0.25; 1.08)	0.24	0.002*
Caregiver HLOE tertiary^b^	0.83 (0.30; 1.37)	0.18	0.002*
SRQ-20 total score	0.07 (0.04; 0.10)	0.19	<0.001*

Model 1: F(11,474) = 3.81 (p < 0.001*), R² = 0.081.

Model 2: F(12,473) = 4.90 (p < 0.001*), R² = 0.110; R² change = 0.029 (*p < 0.001).

*Significance set at p < 0.05.

a WHO stage 1 was the WHO HIV stage category against which other WHO stage categories were analyzed.

b No formal education was the education category against which other education categories were analyzed.

B, the unstandardized regression coefficient or beta; β, the standardized beta; HLOE, highest level of education; SRQ-20, Self-Reporting Questionnaire.

Discrepancy scores were significantly associated with caregiver SRQ-20 scores (*r* = 0.22, *p* < 0.001), and the model was significantly improved when SRQ-20 scores were added to the model (*R*² change = 0.029, *p* < 0.001). Discrepancy scores were significantly associated with caregiver SRQ-20 scores (*B* = 0.07, 95% CI 0.04; 0.10, *p* = 0.011). Caregiver age (*B* = −0.01, 95% CI −0.02; 0.00, *p* = 0.079) and caregiver health status (*B* = −0.13, 95% CI −0.37; 0.11, *p* = 0.273) were no longer significantly associated with discrepancy scores, with a pronounced change for caregiver health status. Similar to adolescents, caregivers with increased psychological distress rated EPs as more severe than children self-rated.

### Sensitivity Analysis With Discrepancy Scores Based on Averaged Severity Scores

Only results that are different (in terms of a change in statistical significance) from the main results are reported.

#### Adolescent BPs

Discrepancy scores based on averaged total severity scores were significantly correlated with discrepancy scores using total severity scores (*r* = 0.986, *p* < 0.001). Adolescent age (*B* = −0.70, 95% CI = −0.15; −0.01, *p* = 0.098) was only trend significantly associated with discrepancy scores.

#### Adolescent EPs

Discrepancy scores based on averaged total severity scores were significantly correlated with discrepancy scores using total severity scores (*r* = 0.937, *p* < 0.001). Discrepancy scores for EPs were significantly associated with adolescent age (*B* = −0.11, 95% CI = −0.20; −0.03, *p* = 0.009) and WHO stage 2 (*B* = 0.44, 95% CI = 0.05; 0.83, *p* = 0.026), such that discrepancy in reporting EPs decreased with advancing adolescent age and caregivers rated EPs as more severe than adolescents if adolescents were in WHO HIV stage 2 as compared to stage 1.

#### Child EPs

Discrepancy scores based on averaged total severity scores were significantly correlated with discrepancy scores using total severity scores (*r* = 0.915, *p* < 0.001). Child age (*B* = −0.10, 95% CI = −0.20; 0.01, p = 0.063) and caregivers having a primary (*B* = 0.40, 95% CI = −0.03; 0.75, *p* = 0.071) level of education were only trend significantly associated with discrepancy scores.

## Discussion

To the best of our knowledge, this is the first study to examine the clinical correlates of informant discrepancy for EBPs among CA-HIV. Older adolescents rated their BPs more severely than their caregivers, whereas caregivers who were employed and HIV negative rated BPs as being more severe than adolescents self-rated. Caregivers rated EPs as more severe than adolescents if the adolescents had WHO HIV stage 4 as compared to stage 1, if the caregiver was also caring for other children, and if the caregiver had a tertiary level of education as compared to no formal education. In the child sample, younger children and older caregivers rated EPs as more severe than their counter informants did. Caregivers rated EPs as more severe than children self-rated if the caregiver’s self-reported health status was poor, and if they had any level of education as compared to no formal education. Informant discrepancy of EBPs was also associated with HIV stigma experienced by adolescents and caregiver psychological distress. Adolescents who reported experiencing HIV-related stigma in the prior year rated their EPs, but not BPs, as more severe than their caregivers rated. Caregivers with greater psychological distress rated all EBPs as more severe than CA-HIV, especially EPs. In summary, caregiver and CA-HIV discrepancy was associated with sociodemographic features of the CA-HIV and their caregivers, HIV disease-related factors, HIV stigma, and caregiver psychological distress and associations varied between EPs and BPs.

Similar to other settings, CA-HIV in this study frequently endorsed experiencing EBPs, with 14.5% of adolescents self-reporting BPs, 28.8% self-reporting EPs, and 36.9% of the children self-reporting EPs. These rates are comparable to what has been found in other studies globally (9, 10, 16, 23, 70). An earlier Ugandan study among HIV-positive adolescents documented higher rates of self-reported psychological distress (51.2%) and substance use (6.1%) (14); however, the adolescents had more advanced HIV and were not receiving ART. Also, in that study, adolescents aged between 13 and 18 years were more likely to be psychologically distressed than those between 10 and 12 years, whereas in our study, rates of self-reported EPs were higher in children than in adolescents. Among CA-HIV, similar to other settings, comorbidity of mental health problems is common (13, 71). In our sample, comorbidity was also frequently reported with 44.0% of adolescents and 42.9% of children with EBPs, endorsing two or more conditions.

Based on previous research with child and adolescent samples, we expected that caregiver and youth agreement would be modest (5, 32, 36, 37). We found generally low agreement between caregivers and CA-HIV, based on symptom severity and symptom count cutoff scores. Although caregiver–adolescent agreement was lower than demonstrated in the YI-4 validation study, it was similar to caregiver–youth agreement in another sample of CA-HIV (7, 33). Agreement between caregivers and children was also of a similar magnitude to the aforementioned study that employed the CI-4 (7). We thus demonstrated similarly low agreement between caregivers and CA-HIV to what has been found in other settings. Meta-analyses have revealed that generally there is greater caregiver–child agreement for externalizing than for internalizing symptoms (32, 37). Similarly, we found that agreement between caregivers and adolescents about the presence of EBPs was significant for BPs, but not EPs. Agreement between caregivers and children regarding the presence of EPs was also significant, suggesting greater agreement between caregivers and children, than between caregivers and adolescents. An earlier meta-analysis also revealed greater agreement between caregivers and younger children, than between caregivers and adolescents, although this association was not demonstrated in a more recent meta-analysis (32, 37). A longitudinal study that evaluated multi-informant reports from childhood into adulthood also found that the agreement between caregiver and youth self-report for internalizing problems decreased as individuals became older, whereas agreement regarding externalizing problems increased with age (36).

It is interesting to note that, in our sample, contrary to our hypothesis, caregivers reported higher rates of EPs than CA-HIV and adolescents self-reported higher rates of BPs than their caregivers. Across various societies, adolescents tend to rate more, and more severe, problems, both internalizing and externalizing, than their caregivers do (36, 51). Other studies of HIV-affected youth have found that caregivers reported more BPs than self-reported by youth (72, 73), and youth reported higher rates of EPs than caregivers (16, 73). Although caregivers reported higher rates of EPs, CA-HIV reported significantly higher rates of SAD, which could be related to context, with SAD occurring in situations where caregivers are often not present; in addition, the internal experience of certain anxiety symptoms may not be evident to caregivers. Generally, agreement has been demonstrated to be higher for observable symptoms than for unobservable symptoms (38). Adolescents also reported higher rates of substance use and conduct problems, which again could reflect the context in which these behaviors occur, as well as adolescents hiding certain unfavorable behaviors from their caregivers. The setting in which discrepancy is being evaluated can also influence the association; for instance, one study found that among clinical samples, both parent- and youth-reported EPs were associated with clinician diagnoses of EPs, but among community samples, only youth self-reported EPs were associated with clinician diagnoses, whereas only parent-reported BPs were associated with clinician diagnoses of BPs among both community and clinic samples (74).

Discrepancy in EP severity ratings decreased with increased child and caregiver age and decreased with increased adolescent age for BPs. Similar to our results, other studies have also found that discrepancies in child BPs decreased with advancing child age (68). The effect of child age on agreement between caregiver and child have, however, been inconsistent and may be related to differences in approach (38). We found that when averaged severity scores were used, associations between discrepancy and child age for EPs, and adolescent age for BPs were no longer significant, whereas adolescent age was significantly associated with discrepancy for EPs. Thus, overall, older CA-HIV rated their EBPs as more severe than caregivers rated them, but this association was of a small magnitude.

Caregivers rated BPs as less severe than adolescents if they themselves were HIV positive or were unemployed. Caregivers rating BPs as less severe than adolescents may reflect caregivers being unaware of adolescent behaviors occurring in contexts outside of the home environment. Parents who are unemployed and HIV positive may be dealing with their own stressors and thus may be less aware of other difficulties faced by adolescents. A systematic review evaluating the effects of HIV-infected caregivers on children in Sub-Saharan Africa reported on a number of studies that noted an association between caregiver HIV and increased EBPs (75). Of note, an earlier Ugandan study found that increased CA-HIV psychological distress was associated with the caretaker being HIV negative (14). Thus, the effect of caregiver HIV status may vary according to the youth’s own status.

Educational attainment of caregivers was associated with increased discrepancy in severity ratings for EPs in both the child and adolescent samples. Caregivers with primary, secondary, or tertiary HLOE rated child EPs as more severe than caregivers with no formal education. In the adolescent sample, this association was demonstrated only for those who had attained a tertiary HLOE as compared to no formal education. In line with these results, a study of CA-HIV found that family characteristics (including caregiver HLOE), largely, were associated with caregiver-rated EBPs, but not with youth self-rated EBPs (7). In contrast, a study of an adolescent forensic sample found that caregiver education had no effect on discrepancy of EBPs (76). An opposite association was demonstrated in a Taiwanese study, where investigators used parental level of education as a proxy of socioeconomic status (SES), and found that parents rated EBPs as less severe than youths if the father had completed tertiary education as compared to not completing secondary education (40). Beyond SES, educational attainment of caregivers may reflect caregiver health/mental health literacy and thus their awareness of EPs.

Adolescents who had attained a WHO HIV stage 4, as compared to stage 1, rated their EPs as less severe than their caregivers did, and when averaged severity scores were used, this was also demonstrated for WHO HIV stage 2. Caregivers of adolescents who had attained a more advanced clinical HIV status may be more concerned about the adolescent’s health status overall due to previous significant health problems. Some studies have demonstrated that more advanced HIV disease may be linked to EBPs in CA-HIV. One study in 81 adolescents found that having a past Centers for Disease Control and Prevention (CDC) class C diagnosis was associated with having at least one prior psychiatric disorder diagnosis and having received prior mental health treatment (18). Another US study of 274 clinically stable CA-HIV found that those with CD4 counts in the lower 50% (<660 cells/mm^3^) were more likely to have caregiver-reported conduct problems (17). In a Malawian study of 562 adolescents, self-rated depression severity was also associated with more severe immunosuppression (based on CD4 count) (19).

Similar to our research, other studies have also found that discrepancies were not related to who the caregiver was (42, 77), although most studies evaluating informant discrepancies have included the mother as caregiver (38). In our sample, caregivers who were also caring for other children rated adolescent EPs as more severe than the adolescents themselves, and caregivers who rated their own general health status lower rated child EPs as more severe than children themselves. Caregivers caring for multiple children may have a higher burden of care, and caregivers with poorer overall health status may also face increased stressors that influence their ratings of CA-HIV mental health status. A study from New York in CA-HIV, aged between 7 and 16 years, utilizing structural equation modeling (SEM), found that caregiver–child stress (including factors such as parent–child relationship problems, caregiver mental health problems, and stressful or negative life events) was associated with both CA-HIV self-reported and caregiver-reported EBPs (78). Other studies have also demonstrated that parents reporting increased caregiving stress rated EBPs as more severe than their children self-reported (42, 76). When caregiver psychological distress was controlled for, however, neither caring for other children nor self-rated health status remained significantly associated with informant discrepancy.

Adolescents who reported experiencing HIV-related stigma in the prior year rated EPs, but not BPs, as more severe than their caregivers did. Of note, when the setting where stigma had been experienced was evaluated, this association was significant for stigma occurring only within the home environment. Other studies in Ugandan adolescents have also demonstrated that HIV-related stigma was associated with increased self-rated mental health problems (27, 29). Adolescents experiencing HIV stigma may develop more EPs; alternatively, adolescents with EPs may interpret events as stigmatizing. Studies have suggested that the pathway between HIV stigma experienced in the community and mental health and adherence outcomes is mediated through internalized stigma (e.g., negative self-views related to HIV) (79). The fact that discrepancy between caregivers and adolescents on EPs was demonstrated largely for stigma experienced in the home environment suggests that familial relational problems may be playing a role in discrepancy, with caregivers not aware of how adolescents are experiencing the home environment as well as the EPs they contend with.

Increased severity of caregiver psychological distress was associated with caregivers rating EPs and BPs as more severe than adolescents self-rated. The association was stronger for EPs than BPs and psychological distress ratings suggestive of possible depression in caregivers was also associated with increased discrepancy of EPs, but not BPs, on bivariate analysis. Our findings are consistent with the majority of the literature (38, 40, 41), although some studies have found that caregiver self-reported mood symptoms were not associated with discrepancy (42). Studies have also demonstrated that maternal anxiety, but not depression, was significantly associated with discrepancy, such that anxious mothers rated their child’s anxiety as more severe than children themselves (80). The SRQ-20 includes symptoms of both anxiety and depression, and we assessed for discrepancy in the severity of CA-HIV EPs combined and thus cannot comment on possible differential associations for anxiety and depression. Increased reporting of EBPs in their children by mothers with depression have been postulated to be due to possible distortion, with depressed mothers over-reporting EBPs, or to reflect accuracy, such that children of depressed mothers have more EBPs and mothers are accurately reporting on those (81). One study found that parental psychological distress was associated with increased parental report of BPs and EPs as well as increased EPs according to parental and child report combined, thus suggesting that parental psychopathology is associated with both increased child EPs and with possible over-reporting of EBPs by parents (82). Regardless of whether discrepancy is due to distortion or an accurate reflection of CA-HIV EBPs, the discrepancy demonstrated still highlights the need for multi-informant reports (41).

### Strengths and Limitations

One of the strengths of the study is the relatively large sample of CA-HIV from a region with a high HIV prevalence. It also includes youth self-report, which is often not assessed in CA-HIV studies, providing unique information on context and how CA-HIV view their own EBPs. This study also considered a broader range of sociodemographic variables than has typically been evaluated in studies examining discrepancy. The assessment instruments adopted for this study were previously used in a large-scale study of CA-HIV from the US (7), thus facilitating cross-cultural comparisons. These measures are also well suited to assess informant agreement as they were designed to have corresponding items and are scored in a similar fashion. Meta-analyses indicate that informant agreement is best evaluated by measures that share the same content, item labeling and scaling (32).

This study also has some notable limitations. We utilized a cross-sectional research design and as such could not determine cause-and-effect relationships. The EBPs reported by caregivers and CA-HIV were not confirmed with a clinical interview, and thus we cannot comment on specificity and sensitivity in this sample. The sample included differed from the sample excluded, and this may have influenced results. Adolescents who were older, living in urban areas, who had a higher socioeconomic index, with caregivers interviewed who were employed and who were not their parents or grandparents were more likely to be excluded. The main reasons these participants were excluded was that caregivers did not complete the CASI-5, and within this context, the differences could be explained by caregivers who were likely unable to complete the assessment as they were working, and that older adolescents were more likely to attend appointments unaccompanied. Children who were residing in urban areas, had a WHO clinical stage 1 or 2, with caregivers interviewed who were younger and HIV negative were more likely to be excluded. The age discrepancy with caregivers is likely due to excluding caregivers who were not older than 17 years; the reasons for the other differences are less apparent. We grouped EBPs together into broad constructs when assessing discrepancy and thus cannot comment on factors associated with discrepancy for individual disorders. The approach we used to determine overall severity of BP and EPs may also have implications. We used total severity scores obtained for disorders to compute discrepancy scores, which may have weighted total severity towards disorders with more symptoms. However, sensitivity analysis with discrepancy scores determined with averaged severity scores mostly provided the same results, with some differences noted. The differences were of a small magnitude, such that significant associations became trend significant and vice versa. The strength of association for the factors that differed according to approach used to determine severity scores was thus likely small. Future studies can perform more in-depth analysis evaluating agreement and discrepancy for individual disorders as it is beyond the scope of this manuscript. Although we noted receipt of ART, data were not collected on specific ARV regimens. Lastly, we did not correct for multiple comparisons (and type I error) and thus our results should be considered exploratory.

### Conclusions

Similar to other studies, we demonstrate that CA-HIV commonly experience EBPs, whether based on caregiver or youth self-report. Furthermore, CA-HIV may be prone to develop more severe psychopathology as reflected by higher rates of psychiatric hospitalizations and increased rates of prior psychotropic and behavioral treatments (35, 83). Mental health problems can have serious detrimental consequences in individuals with HIV; for instance, higher mortality rates were reported for HIV-infected individuals with comorbid psychiatric and substance use disorders than those without (84). In the aforementioned study, the mortality risk was lower in those who had received treatment for EBPs, highlighting the importance of screening and timely intervention. Although mental health outcomes have not always been linked to adherence problems in individual studies, a systematic review identified mental health as a factor influencing adherence, particularly when other risk factors are also present (85). Despite the burden of mental health problems in CA-HIV, there is a dearth of appropriate mental health services, especially in resource-limited settings (15).

To our knowledge, this is the first study to evaluate informant discrepancy of EBPs in CA-HIV. Discrepancy between caregivers and CA-HIV was greater if caregivers were unemployed and had no formal education. These results suggest that socioeconomic status may influence the discordance between caregivers and CA-HIV regarding the presence of EBPs. Although many studies have not found an association between socioeconomic status and informant discrepancies, a meta-analysis demonstrated that agreement between mothers and fathers on internalizing and externalizing problems was lower for children of low socioeconomic status (38, 86). Of note, some HIV-related variables were associated with discrepancy (i.e., caregivers rating more severe BPs than adolescents if they were HIV negative and more severe EPs if the child had a history of more advanced clinical HIV). These findings suggest that HIV disease-related factors may influence caregiver CA-HIV discrepancy regarding EBPs. HIV stigma was associated with discrepancy, yet again highlighting the negative impact of HIV stigma as well as the importance of the context in which it is experienced. In addition to HIV-related stigma, mental-health-related stigma can be another barrier to CA-HIV receiving mental health care (15). We assessed only for adolescent perceived interpersonal HIV stigma, and future investigations utilizing broader constructs related to stigma, such as internalized HIV stigma, HIV disclosure stigma, stigma in health care services, mental health stigma, and stigma experienced by caregivers, are warranted. Consistent with prior research (38, 40, 41), we also demonstrated that caregivers with increased psychological distress rate the EBPs of CA-HIV as more severe than youths self-rated.

Informant discrepancies have been linked to poorer youth outcomes (42, 43). Fewer discrepancies between caregivers and youths predict improved treatment engagement and outcomes (44, 87, 88). Informant discrepancy can, therefore, be considered in treatment planning and may contribute to improved treatment outcomes, further highlighting the value of evaluating informant discrepancy in CA-HIV (42, 89). Our study supports the general consensus that mental health screening and assessments, including CA-HIV self-report, should be integrated into routine care and multilevel psychosocial and family-based interventions are needed to support CA-HIV and their caregivers (9).

## Ethics Statement

The study was conducted in accordance with the Declaration of Helsinki and ethical approval was obtained from the Uganda Virus Research Institute’s Research and Ethics Committee, the Ethics Committee of the London School of Hygiene and Tropical Medicine, and the Uganda National Council of Science and Technology. Eligible study participants provided written informed consent (caregiver) and assent (CA-HIV) after explanation of the study objectives and procedures. No CA-HIV were enrolled without their assent and all participants were informed that they could withdraw without prejudice at any time. In the majority of cases, the parents provided informed consent for participation of the CA-HIV, but in cases where the primary caregivers were not parents, the guardians of the CA-HIV provided the informed consent.

## Author Contributions

EK, JL, KG, and VP contributed to the concept and design of the study. Data collection was done by EK, JL, and RM. LH performed statistical data analysis and wrote the paper. All authors contributed to revising the manuscript and have read and approved the submitted version.

## Funding

This work was supported by an MRC/DFID African Leadership Award to Eugene Kinyanda, Number MR/L004623/1. Leigh van den Heuvel was supported in this work by a NIH-Fogarty Project Titled: “Pittsburgh-Stellenbosch University AIDS-comorbidities Training Research Programme (Pitt-SU AICoTRP; Award to Jean B. Nachega and Soraya Seedat)” and her work reported herein was made possible through funding by the South African Medical Research Council through its Division of Research Capacity Development under the SAMRC CLINICIAN RESEARCHER (M.D PHD) SCHOLARSHIP PROGRAMME from funding received from the South African National Treasury. The content hereof is the sole responsibility of the authors and do not necessarily represent the official views of the SAMRC or the funders. Soraya Seedat is supported by the South African Research Chairs Initiative in PTSD funded by the Department of Science and Technology and the National Research Foundation.

Funders have played no role in the study design, data collection, analysis, and interpretation and in writing the manuscript.

## Conflict of Interest Statement

KG is a shareholder in Checkmate Plus and publisher of the CASI-5, YI-4, and CI-4.

The remaining authors declare that the research was conducted in the absence of any commercial or financial relationships that could be construed as a potential conflict of interest.

The reviewer JA declared a shared affiliation, with no collaboration, with one of the authors JN to the handling editor.
